# Association of Obesity and Type 2 Diabetes Mellitus With Periodontitis: A Cross-Sectional Study

**DOI:** 10.7759/cureus.68779

**Published:** 2024-09-06

**Authors:** Garima Asthana, Pooja Palwankar, Ruchi Pandey

**Affiliations:** 1 Periodontology, Manav Rachna Dental College, School of Dental Sciences, Manav Rachna International Institute of Research and Studies (MRIIRS), Faridabad, IND

**Keywords:** anthrpometry, bioelectrical impedance analysis, bmi, diabetes, obesity, periodontitis

## Abstract

Background

The relationship between obesity and type 2 diabetes mellitus (T2DM) is inevitable. The increase in the occurrence of obesity all around the globe has proportionally increased the occurrence of comorbidities. Periodontitis is a multifactorial inflammation of the periodontium attributed to dysbiosis in the subgingival microflora which may ultimately result in tooth loss. A triad between T2DM, periodontitis, and obesity is ascertained.

Aim

The present study focuses on investigating the association of obesity and T2DM with periodontal health.

Methodology

This cross-sectional study was conducted for a period of six months (September 2022 to February 2023) on 181 subjects, as per the sample size calculated by the statistician, who were previously diagnosed with T2DM and were either obese or overweight. The glycemic control was assessed on the basis of HbA1c values of the subjects. The subjects underwent bioelectrical impedance analysis along with an anthropometric examination. Full mouth examination including bleeding on probing, pocket probing depth, clinical attachment level (CAL), and oral hygiene index-simplified (OHI-S) was also checked to assess the status of periodontal health, and periodontitis was classified according to the new classification of 2017.

Results

The obtained data was statistically analyzed and p-value≤0.05 was considered statistically significant. The maximum prevalence of Stage III Grade C Periodontitis (34.73%) was observed in the diabetic obese group than in the diabetic overweight group. The overall anthropometric variable, abdominal circumference, waist-hip ratio, and basal metabolic index (BMI) were higher in the obese group as they displayed poor glycemic control. BMI and CAL also showed a positive correlation.

Conclusion

A significant association between obesity and T2DM with periodontitis was confirmed by this study. Hence, a syndemic approach needs to be formulated by the medical fraternity in collaboration with dental surgeons for the effective management of this triad.

## Introduction

The global amelioration in technology and urbanization has led to decreased physical activity in life and unhealthy eating habits have led to overweight or being obese, thereby adversely affecting the quality of life. According to the reports of World Obesity Atlas 2024, the projected increase in the global prevalence of obesity in adults with high body mass index (BMI) is 1.25 billion by 2030 as compared to 0.81 billion in 2020 [1]. Being overweight is a condition characterized by excessive deposit of adipose tissues while obesity is a chronic convoluted disease that is caused due to excessive fat deposition and also increases the risk of type 2 diabetes mellitus (T2DM), cancer, cardiovascular diseases, and various other systemic diseases. Obesity is not only limited to adults but also affects children. India has been enlisted in the top 20 countries of the world where there is a rapid increase in the proportion of children living with a high BMI as estimated from 2000 to 2016 [1].

BMI is the gold standard for assessing overweight or obesity, it is the ratio of weight in kilograms (kgs) to height in square meters (m^2) multiplied by 703 [2]. However, other parameters such as abdominal circumference and waist-hip ratio (WHR) may also be helpful in diagnosing obesity. Bioelectrical impedance analysis (BIA) is one of the extremely favored methods for the assessment of body composition [3]. It is based on the principle of differential rate of electric current flow through the body based on its composition. BIA is a valuable tool for a healthcare professional as it indicates obesity and imbalance in body composition at an early stage thereby reversing the progression of the disease to state of health.

Diabetes mellitus is a chronic metabolic disorder which not only affects various organs of the body but also has a major impact on the oral health. A Global Burden of Disease published in the Lancet in the year 2023 revealed that in 2021, 529 million individuals were living with diabetes globally indicating a worldwide prevalence rate of 6.1%. Considering the current trend of increase in the rate of diabetes, it has been projected that by 2050 more than 1.31 billion people will be suffering from the disease [4]. In the global scenario, South Asia is the home of about a quarter of the total population. A study conducted by the Indian Council of Medical Research- India Diabetes (ICMR-INDIAB) in 2023 under the Ministry of Health and Family Welfare (MoHFW) observed that 26.6% of the Indian population was suffering from dysglycemia accounting for about 236 million people including both prediabetes and diabetes patients [5].

The relationship between obesity and T2DM is inevitable. The increase in the occurrence of obesity all around the globe has proportionally increased the occurrence of comorbidities. Disproportionate accumulation and deranged expansion of adipose tissue in an obese body disturb homeostasis thereby by encouraging insulin resistance. This further compromises the host immune response by means of systemic inflammation which causes dysfunctioning of β-cells and causes persistent elevation of blood glucose levels [6].

A triad between T2DM, periodontitis, and obesity is ascertained. Periodontitis is a multifactorial inflammation of the periodontium attributed to dysbiosis in the subgingival microflora which may ultimately result in tooth loss. The new classification of periodontal and peri-implant diseases proposed by the World Workshop of Periodontology 2017 periodontitis has been classified into four stages and three grades. The new classification has also included diabetes and smoking as grade modifiers to assess the progression of periodontal disease [7,8].

The present study focuses on investigating the association of obesity and T2DM with periodontal health.

## Materials and methods

Subjects

After obtaining ethical clearance from the institutional ethics committee of Lady Hardinge Medical College, New Delhi (LHMC/IEC/2022/PG Thesis/48) and informed consent, 200 subjects reporting to the diabetic clinic of the Department of Medicine, Lady Hardinge Medical College, New Delhi were screened for the study. Subjects aged between 18 and 70 years, previously diagnosed with T2DM, BMI ≥ 25kg/m^2^, and dentate patients with a minimum of 20 teeth in the oral cavity were included. Pregnant and lactating women, all forms of tobacco users, Type 1 diabetes mellitus patients or any other systemic disease, subjects on medication that could affect periodontal condition, fixed orthodontic treatment, and history of periodontal treatment in the last three months were excluded. A total of 181 subjects were recruited for the study which included 41 males (22.6%) and 140 females (77.3%) as per the sample size calculated by the statistician using OpenEpi, Version 3, open-source calculator-SSMean software.

Following a detailed case history and clinical examination of the subjects, bioimpedance analysis was performed using a bioimpedance analyzer (ACCUNIQ BC300, SELVAS Healthcare, South Korea) to assess the body fat composition.

Measurement of obesity

Anthropometric data including weight, height, BMI, abdominal circumference (AC), and WHR was recorded in light clothing by trained personnel on a bioimpedance analyzer. Individuals with a BMI between 23 and 24.9 kg/m^2^ were overweight and BMI above 25 kg/m^2^ were considered obese [9]. Males with AC ≥ 102 cm and females > 88 cm were classified as obese [10]. Similarly, WHR ≥ 0.85 indicates obesity in women and over 0.90 in men according to WHO [11].

Measurement of control of T2DM

The diabetic status was assessed on the basis of HbA1c levels. Subjects with HbA1c levels less than 7% were categorized as well-controlled diabetics while those whose values were above 7% depicted poor control of blood sugar levels according to the American Diabetes Association [12].

Assessment of periodontal health

All the participants were subjected to full-mouth periodontal examination to assess the periodontal parameters. Bleeding on probing (BOP), pocket probing depth (PPD), clinical attachment level (CAL), and oral hygiene index-simplified (OHI-S) using UNC-15 periodontal probe (Hu-Friedy) at six sites per tooth was assessed and diagnosis was made according to 2017 classification [13].

Statistical analysis

The Chi-square test was used for analyzing demographic data. An independent t-test was incorporated for intergroup comparison and one-way ANOVA to compare the variances across means of different groups. Pearson’s correlation was used to find the association between BMI and CAL. All the analysis was carried out with the help of IBM SPSS Statistics for Windows, Version 21 (Released 2012; IBM Corp., Armonk, New York, United States) and a p-value < 0.05 was considered statistically significant.

## Results

Demographic details and oral hygiene characteristics

The study consisted of 181 T2DM subjects including 13(18.8%) males and 56(81.2%) females aged between 18 and 45 years. Twenty-eight (25%) males and 84 (75%) females were aged between 45 and 70 years. The mean age of females was 49±10.7 years and in males, it was calculated as 49±10.60 years. It was observed that 80.7% of the population maintained a fair oral hygiene and used toothbrushes to clean their teeth. Fifty-nine (32.6%) subjects out of 181 brushed their teeth twice daily and 122 (67.4%) subjects brushed once daily.

Anthropometric data based on bioimpedance analysis

Anthropometric data analyses showed that 95 (52.4%) were overweight and 86 (47.5%) were obese. The mean BMI in the overweight group was 28.66±2.80 kg/m^2^ and in the obese group was 32.44±5.21 kg/m^2^ which was statistically significant (p<0.001). Similarly, the mean abdominal circumference in overweight individuals was 88.35±7.94 cm and 94.21±8.97 cm in obese individuals, and the WHR in overweight individuals was 0.90±.05cms and 0.95±.06 in obese individuals. The difference in the abdominal circumference and WHR in both overweight and obese individuals was statistically significant (Table 1).

**Table 1:** Anthropometric Data based on Bioimpedance Analysis SD: Standard Deviation; BMI: Basal Metabolic Index; *Chi‐square test; significance at p< 0.05

Variables	Obesity Analysis ( Mean±SD)		p-Value
	Overweight (n=95)	Obese (n=86)	
BMI	28.66±2.80	32.44±5.21	*<0.001, S
Abdominal Circumference	88.35±7.94	94.21±8.97	*<0.001, S
Waist-Hip Ratio	0.90±.05	0.95±.06	*<0.001, S

Periodontal health status according to obesity

The mean PPD in the obese group was 6.81±2.01 mm and 5.72±1.25 mm in non-obese group. Similarly, the CAL was significantly higher in the obese group, p<0.001. Both the groups displayed poor oral hygiene status on the basis of OHI-S results. Results indicated that glucose control in the obese group (10.0±2.03%) compared to the non-obese group (8.24±1.5mm) was statistically significant (Table 2). BOP was found to be positive in all 181 subjects.

**Table 2:** Periodontal Status According to Obesity Based on BMI SD: Standard Deviation; PPD: Pocket Probing Depth; CAL: Clinical Attachment Level; OHI-S: Oral Hygiene Index- Simplified; **Independent* t-test* ; significance at p<0.05

Variables	Obesity Analysis ( Mean±SD)		p-Value
	Overweight (n=95)	Obese (n=86)	
PPD (in mm)	5.72±1.25	6.81±2.01	**<0.001, S
CAL (in mm)	5.72±3.20	8.34 ±2.93	**<0.001, S
OHI-S Score	3.50±0.87	4.04±0.89	**<0.001, S

**Figure 1:**
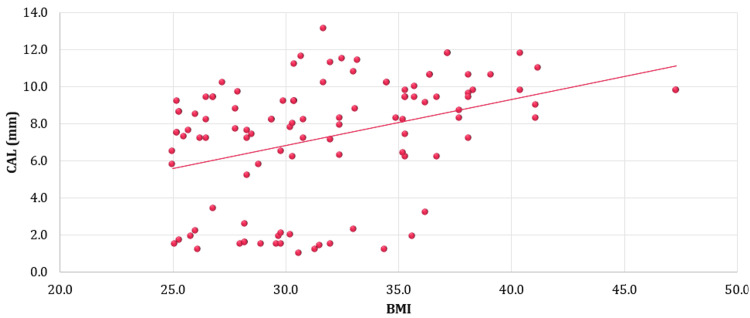
Correlation between BMI and CAL BMI: Basal Metabolic Index; CAL: Clinical Attachment Level; Pearson's correlation

A positive correlation was seen between BMI and CAL (Figure 1).

Prevalence of periodontitis in obese and overweight individuals

It was observed that a maximum of 60 (70%) patients in the obese group suffered from Stage III grade C periodontitis, whereas 33 (34.73%) subjects in the overweight group also suffered from Stage III grade C periodontitis (Table 3).

**Table 3:** Occurrence of periodontitis in obese and overweight individuals One-wayANOVA test

			Overweight (n=95)		Obese (n=86)	
			Grade B	Grade C	Grade B	Grade C
Periodontitis	Stage I	n(%)	1(0.01)	4(0.04)	1(0.01)	1(0.01)
	Stage II	n(%)	22(23.15)	22(23.15)	1(0.01)	23(26.74)
	Stage III	n(%)	13(13.7)	33(34.73)	1(0.01)	60(70)
Total		n(%)	36(37.9)	59(62.1)	2(2.3)	84(97.7)

Stage III periodontitis had the maximum prevalence in both the groups. However, the number of individuals suffering from Stage 3 periodontitis was significantly higher in the obese group, 61(33.7%) as compared to 46 (25.41%) subjects in the overweight group. 

## Discussion

The current research is a novel study to find the association of periodontal disease classified on the basis of American Association of Periodontology (AAP) classification 2017 in previously diagnosed T2DM patients who were either overweight or obese.

T2DM is emerging as a pandemic with India leading the race and has been declared the world’s capital of diabetes with an estimated diabetic population of 80 million by 2030 [14]. HbA1c is a validated diagnostic method for individuals who are at risk of developing diabetes mellitus. According to The Diabetes Chronic Complications Trial (DCCT) and United Kingdom Prospective Diabetes Study (UKPDS), HbA1c was demonstrated to be a beneficial test for delaying and reducing the complications of diabetes mellitus [15]. A syndemic relationship occurs between diabetes mellitus, obesity, and periodontal disease. According to the WHO fact sheet, approximately 44% of obese individuals suffer from diabetes and it has been projected that about three million will be affected by obesity by 2025 [16]. Various methods are used to assess the obesity of an individual like BMI, anthropometry, bioelectrical impedance analysis, etc. However, the BMI measure has its own shortcomings as obesity is a multifactorial disease. Therefore, BIA may be used in conjunction with BMI to attain accurate measures of obesity. In this study, BIA and anthropometry were used along with BMI for more precise results.

It is not only the etiopathogenesis of non-communicable diseases (NCDs) that affects periodontal health, but an unhealthy lifestyle may also be considered as one of the attributes of poor oral health due to reduced physical activity, lethargy, and psychosocial stress. However, the effect of elevated adipokines on insulin resistance cannot be ignored as this mechanism alters the pathogenicity of oral microflora. Our study revealed significantly higher values of BMI, AC, and WHR in the obese group with diabetes. The Hba1C levels were significantly higher in the population indicating poor diabetes control. These results are in accordance with the study by Saberi-Karimian et al. wherein they concluded that abdominal circumference was the most important parameter in predicting the risk of T2DM in obese patients [17]. BOP was positive in all the subjects which was suggestive of ongoing inflammation in gingiva progressing to periodontal tissues. The obese group displayed a poor oral health status as well as poorer periodontal health by significantly elevated values of PPD, CAL, and OHI-S scores as compared to overweight individuals. A positive correlation was observed between BMI and CAL showing an increased risk of periodontal breakdown in obese individuals. These results were similar to other studies which showed that CAL and PPD in the obese group of diabetic patients indicated a higher risk of development of periodontal disease in the obese group [18-20].

The plausible association between BMI and CAL could be attributed to increased production of adipokines that alter the balance between pro-inflammatory cytokines (IL-1, IL-6, TNF-α, etc.) and anti-inflammatory cytokines (visfatin, leptin, resistin, and adiponectin) thereby shifting the immune response against periodontal pathogens which ultimately leads to increased susceptibility to periodontal disease [21]. Obesity may also affect the periodontal status of an individual via insulin resistance [22], altered virulence of a specific periodontopathogen [23], and alteration in the microcirculation and blood supply of the gingiva [24]. In this study, periodontal parameters according to the three measures of obesity: BMI, AC, and WHR were significantly correlated with PPD and CAL in the obese group and increased abdominal circumference. These results are in accordance with the study performed by Pham and Tran where they observed a significant association between obesity, T2DM, and periodontitis [25]. This result is also in accordance with the study done by Saberi-Karimian et al. [17].

A hyperglycemic state causes elevation of AGEs which in turn causes an increase in the concentration of pro-inflammatory mediators leading to increased tissue destruction, bone resorption, and impaired tissue repair [26].

Furthermore, in this study authors have graded periodontitis on the basis of indirect evidence of disease progression (case phenotype) and HbA1c, a grade modifier. An overall prevalence of Stage II grade C (70%) periodontitis was observed in the obese diabetic group as they displayed poor glycaemic controls. Similar results have been obtained in a study done by Jia et al. [27] and Sheokand et al. [28].

In our study, a strong association between T2DM and obesity with periodontitis was found which accepts the alternate hypothesis. It was found that individuals with uncontrolled diabetes and obesity displayed an aggravated form of periodontitis. Considering the rapid progression of the triad of these non-communicable diseases “4th High-level Meeting of the United Nations General Assembly on the Prevention and Control of NCDs (2025)”, it has been proposed to implement policies to drastically reduce the exposure of individuals and population to the risk factors for NCDs [29]. However, as already known, periodontitis is a multifactorial disease that shares a systemic relationship with various diseases but the progression of the disease is primarily attributed to the accumulation of dental plaque that majorly consists of microorganisms. Therefore, this triad requires a syndemic approach which would include maintenance of good oral hygiene, glycemic control as well as lifestyle and diet modifications. This study gave an insight into the interrelationship between the three diseases but has its own limitations.

The study was carried out at a tertiary care medical center with a small sample size. Therefore, cause-effect relationships cannot be depicted as the sample size is not a representative of the general population. Also, some of the major confounding factors like hypertension, osteoporosis, or rheumatism which may have a relationship with periodontal disease were not taken into consideration. Therefore, more longitudinal studies are required along with radiographic evaluation of the subjects to confirm this relationship on a larger sample size with equal gender distribution.

## Conclusions

A positive correlation was found between obesity, T2DM, and periodontal diseases. Although this relationship has been reported in other studies, this is the first study where association of periodontitis with T2DM and obesity has been compared between obese and overweight diabetic subjects using the newer classification of periodontitis proposed by AAP in 2017.

Therefore, this study highlights the clinical evidence of a relationship between the three NCDs and the importance of assessment of glycemic tests and anthropometric measurements of the subjects along with oral examination. A collaborative treatment approach of the medical and dental fraternity in the management of T2DM and obesity along with management of periodontal diseases will provide a holistic treatment to the patients as treatment of T2DM and obesity have a direct impact on periodontal health which in turn affects the quality of life of an individual.
